# Supplementary material for: Enhanced cybersecurity threat detection using novel tri-metaheuristic loss functions in generative adversarial networks with adaptive attention preservation for network traffic augmentation
Source: Sci Rep. 2026 Apr 12;16:12074. doi: 10.1038/s41598-026-46375-3 (PMC13076765; doi:10.1038/s41598-026-46375-3)
Supplement: Supplementary file 1 — Supplementary Material 1 [file 41598_2026_46375_MOESM1_ESM.docx]

**Table A1: Hyperparameter Configuration for Tri-Metaheuristic GAN Framework**

| Category | Parameter | Value | Search Range | Optimization Method |
| --- | --- | --- | --- | --- |
| Optimization | Generator Learning Rate | 3×10⁻⁴ | [1×10⁻⁵, 5×10⁻⁴] | Bayesian optimization |
|  | Discriminator Learning Rate | 1×10⁻⁴ | [5×10⁻⁵, 2×10⁻⁴] | Bayesian optimization |
|  | Adversarial Learning Rate | 5×10⁻⁵ | [1×10⁻⁵, 1×10⁻⁴] | Bayesian optimization |
|  | Optimizer | AdamW | [Adam, AdamW, RAdam] | Grid search |
|  | Beta₁ | 0.5 | [0.3, 0.9] | Grid search |
|  | Beta₂ | 0.999 | [0.99, 0.9999] | Fixed |
|  | Weight Decay | 1×10⁻⁵ | [0, 1×10⁻⁴] | Bayesian optimization |
|  | Gradient Clipping | 1.0 | [0.5, 2.0] | Grid search |
| Training Schedule | Total Epochs | 150 | [100, 200] | Sustainability-aware |
|  | Warmup Epochs | 15 | [10, 20] | Grid search |
|  | Metaheuristic Start | 16 | [11, 25] | Grid search |
|  | Fine-tuning Start | 101 | [80, 120] | Grid search |
|  | Early Stopping Patience | 25 | [15, 35] | Sustainability-aware |
|  | Batch Size | 128 | [64, 256] | Memory optimization |
|  | Gradient Accumulation | 2 steps | [1, 4] | Effective batch tuning |
| Loss Weights | α (Firefly Luminosity) | 0.35 | [0.2, 0.5] | Bayesian optimization |
|  | β (Firefly Attraction) | 0.30 | [0.2, 0.4] | Bayesian optimization |
|  | γ (Firefly Absorption) | 0.35 | [0.2, 0.5] | Bayesian optimization |
|  | δ (Jellyfish Ocean Current) | 0.32 | [0.2, 0.5] | Bayesian optimization |
|  | ε (Jellyfish Swarm) | 0.38 | [0.2, 0.5] | Bayesian optimization |
|  | ζ (Jellyfish Time Control) | 0.30 | [0.2, 0.4] | Bayesian optimization |
|  | η (Mantis Strike) | 0.35 | [0.2, 0.5] | Bayesian optimization |
|  | θ (Mantis Vision) | 0.33 | [0.2, 0.5] | Bayesian optimization |
|  | κ (Mantis Territory) | 0.32 | [0.2, 0.4] | Bayesian optimization |
|  | λ (Attention Preservation) | 8.0 | [1.0, 15.0] | Bayesian optimization |
|  | μ (Adversarial Weight) | 1.0 | [0.5, 2.0] | Fixed baseline |
| Energy-Aware | Energy Budget (kWh) | 80.0 | [60, 100] | Sustainability target |
|  | Carbon Budget (kg CO₂) | 35.0 | [25, 50] | Climate commitment |
|  | Efficiency Threshold | 1.8× | [1.5×, 2.5×] | Performance-energy balance |
|  | Renewable Scheduling | Enabled | [Enabled, Disabled] | Grid availability |

**Table A2: Comprehensive Evaluation Metrics for Security and Sustainability Assessment**

| Category | Metric | Measurement | Target/Baseline |
| --- | --- | --- | --- |
| Detection Performance | Accuracy | (TP + TN) / (TP + TN + FP + FN) | > 98% / 93.45% baseline |
|  | Precision | TP / (TP + FP) | > 97% / 91.23% baseline |
|  | Recall (Sensitivity) | TP / (TP + FN) | > 97% / 89.67% baseline |
|  | F1-Score | $2\cdot\frac{Prec\cdot Rec}{Prec+Rec}$ | > 0.97 / 0.90 baseline |
|  | False Positive Rate | FP / (FP + TN) | < 2% / 8.77% baseline |
|  | False Negative Rate | FN / (TP + FN) | < 1% / 10.33% baseline |
|  | AUC-ROC | Integral of TPR vs FPR | > 0.99 / 0.94 baseline |
| Adversarial Robustness | Robust Accuracy (FGSM) | $Standard accuracy on x_{adv}$ | > 94% / 78% baseline |
|  | Robust Accuracy (PGD) | 40-iteration PGD evaluation | > 93% / 72% baseline |
|  | Robust Accuracy (C&W) | Optimization-based attack | > 92% / 68% baseline |
|  | Perturbation Sensitivity | Δ Accuracy / Δ ε | < 8% per 0.1 ε / 15% baseline |
| Energy Efficiency | Training Energy (kWh) | $Hardware measurement via\frac{NVML}{\mu Prof}$ | < 80 kWh / 128.4 kWh baseline |
|  | Inference Energy (mJ) | Batch inference energy / batch size | < 25 mJ / 42 mJ baseline |
|  | Energy Efficiency Ratio | (Accuracy ×100) / Training Energy | > 1.23 / 0.73 baseline |
|  | FLOPs Reduction | $\frac{\left( FLOPs_{baseline}- FLOPs_{proposed} \right)}{FLOPs_{baseline}}$ | > 35% / 0% baseline |
|  | Memory Efficiency | Maximum GPU memory during training | < 45 GB / 68 GB baseline |
| Carbon Footprint | Training Carbon (kg CO₂) | Energy × carbon intensity × PUE | < 35 kg / 67.3 kg baseline |
|  | Inference Carbon (g CO₂) | (Energy/sample × 10^6) × intensity | < 15 g / 28 g baseline |
|  | Carbon Efficiency | Accuracy / Carbon Emissions | > 2.82 / 1.39 baseline |
|  | Renewable Energy Fraction | Renewable kWh / Total kWh | > 55% / 23% baseline |
| Training Efficiency | Convergence Speed | Monitoring during training | < 90 epochs / 137 epochs baseline |
|  | Training Time (hours) | Including renewable scheduling | < 23 hours / 32 hours baseline |
|  | Parameter Efficiency | Total trainable parameters | < 45M / 67M baseline |
| Generalization | Cross-Dataset Accuracy | Direct transfer without retraining | > 87% / 76% baseline |
|  | Fine-Tuning Efficiency | 2-5 hour fine-tuning | > 93% / 84% baseline |
|  | Zero-Day Detection | Hold-out unseen attack families | > 89% / 71% baseline |

**Table A3: Comprehensive Ablation Study Results**

| Configuration | Firefly | Jellyfish | Mantis Shrimp | Attention | Energy-Aware | Accuracy (%) | PSNR (dB) | F1-Score | Training Energy (kWh) | Robust Acc ε=0.3 (%) |
| --- | --- | --- | --- | --- | --- | --- | --- | --- | --- | --- |
| Baseline GAN | ✗ | ✗ | ✗ | ✗ | ✗ | 84.23 ± 2.15 | 24.56 ± 1.23 | 0.830 | 128.4 ± 9.8 | 61.23 ± 3.45 |
| + Firefly Only | ✓ | ✗ | ✗ | ✗ | ✗ | 88.46 ± 1.78 | 28.34 ± 0.98 | 0.878 | 118.7 ± 8.9 | 68.45 ± 3.12 |
| + Jellyfish Only | ✗ | ✓ | ✗ | ✗ | ✗ | 87.90 ± 1.85 | 27.21 ± 1.05 | 0.871 | 121.3 ± 9.2 | 66.78 ± 3.28 |
| + Mantis Only | ✗ | ✗ | ✓ | ✗ | ✗ | 87.12 ± 1.92 | 26.45 ± 1.12 | 0.865 | 123.8 ± 9.5 | 82.34 ± 2.45 |
| Firefly + Jellyfish | ✓ | ✓ | ✗ | ✗ | ✗ | 92.67 ± 1.34 | 30.89 ± 0.87 | 0.923 | 108.5 ± 8.2 | 74.56 ± 2.89 |
| Firefly + Mantis | ✓ | ✗ | ✓ | ✗ | ✗ | 91.89 ± 1.42 | 29.78 ± 0.92 | 0.915 | 111.2 ± 8.5 | 86.23 ± 2.12 |
| Jellyfish + Mantis | ✗ | ✓ | ✓ | ✗ | ✗ | 91.34 ± 1.48 | 29.12 ± 0.95 | 0.910 | 113.6 ± 8.7 | 85.45 ± 2.23 |
| Triple without Attention | ✓ | ✓ | ✓ | ✗ | ✗ | 95.78 ± 0.98 | 32.45 ± 0.72 | 0.956 | 98.3 ± 7.4 | 91.67 ± 1.45 |
| Triple without Energy-Aware | ✓ | ✓ | ✓ | ✓ | ✗ | 98.45 ± 0.48 | 33.12 ± 0.68 | 0.984 | 124.7 ± 9.1 | 94.89 ± 0.98 |
| Full Proposed Framework | **✓** | **✓** | **✓** | **✓** | **✓** | **98.73 ± 0.41** | **33.67 ± 0.65** | **0.987** | **76.8 ± 5.4** | **95.67 ± 0.82** |
| Individual Firefly Contribution | — | — | — | — | — | +4.23% | +3.78 dB | +0.048 | -9.7 kWh | +7.22% |
| Individual Jellyfish Contribution | — | — | — | — | — | +3.67% | +2.65 dB | +0.041 | -7.1 kWh | +5.55% |
| Individual Mantis Contribution | — | — | — | — | — | +2.89% | +1.89 dB | +0.035 | -4.6 kWh | +21.11% |
| Attention Block Contribution | — | — | — | — | — | +2.95% | +0.67 dB | +0.031 | +25.4 kWh | +3.22% |
| Energy-Aware Contribution | — | — | — | — | — | +0.28% | +0.55 dB | +0.003 | -47.9 kWh | +0.78% |

Table A4: Component Contribution by Attack Type on NSL-KDD Dataset

| Component | DoS (%) | Probe (%) | R2L (%) | U2R (%) | Normal (%) | Analysis |
| --- | --- | --- | --- | --- | --- | --- |
| Class rebalancing | +1.8 | +2.1 | +8.9 | +12.3 | +0.4 | Greatest benefit for rare classes |
| Standard augmentation | +1.2 | +1.5 | +4.2 | +5.8 | +0.3 | Moderate benefit for minority |
| Distribution alignment | +2.1 | +2.4 | +3.1 | +3.5 | +1.8 | Uniform improvement |
| Clustering loss | +1.4 | +2.8 | +2.3 | +2.1 | +1.2 | Strong for Probe patterns |
| Multi-scale preservation | +1.8 | +1.2 | +1.5 | +1.8 | +0.9 | Benefits signature-rich attacks |
| Adversarial robustness | +1.1 | +1.3 | +2.4 | +2.9 | +0.8 | Critical for evasive attacks |
| Attention preservation | +0.4 | +0.5 | +1.2 | +1.4 | +0.3 | Benefits feature-sensitive classes |
| Total improvement | +9.8 | +11.8 | +23.6 | +29.8 | +5.7 | Largest gains for minority classes |

Table A5: Statistical Significance Analysis of Component Contributions

| Component | Mean Δ | 95% CI | p-value (paired t-test) | Cohen's d | Significance |
| --- | --- | --- | --- | --- | --- |
| Distribution alignment loss | +2.33% | [2.01, 2.65] | < 0.0001 | 1.89 | Highly significant |
| Clustering discriminator loss | +1.56% | [1.28, 1.84] | < 0.0001 | 1.45 | Highly significant |
| Multi-scale preservation loss | +1.22% | [0.98, 1.46] | < 0.0001 | 1.21 | Highly significant |
| Adversarial robustness loss | +1.11% | [0.89, 1.33] | < 0.0001 | 1.15 | Highly significant |
| Attention preservation loss | +0.56% | [0.38, 0.74] | 0.0003 | 0.78 | Significant |
| Energy-aware optimization | +0.22% | [0.08, 0.36] | 0.0124 | 0.34 | Significant |
| Component synergy | +0.39% | [0.21, 0.57] | 0.0018 | 0.52 | Significant |

**Table A6: Theoretical Computational Complexity Comparison**

| Component/Method | Time Complexity | Space Complexity | Dominant Operations |
| --- | --- | --- | --- |
| Proposed Framework Components |  |  |  |
| Feature importance loss (Eq. 4) | O(Bd) | O(Bd) | Softmax attention computation |
| Distribution alignment loss (Eq. 9) | O(Bd) | O(B) | Discriminator forward pass |
| Gradient regularization loss (Eq. 12) | O(Bd²) | O(Bd) | Second-order gradient computation |
| Adversarial discrimination loss (Eq. 15) | O(Bd) | O(B) | Hinge loss with ReLU |
| Clustering discriminator loss (Eq. 17) | O(B²dₑ) | O(B²) | Pairwise distance computation |
| Curriculum scheduling loss (Eq. 18) | O(Bd) | O(B) | Linear interpolation |
| Perturbation-aware loss (Eq. 26) | O(TBd) | O(Bd) | T iterations of PGD |
| Multi-scale preservation loss (Eq. 28) | O(SBd) | O(SBd) | S-scale wavelet decomposition |
| Diversity regularization loss (Eq. 40) | O(B²dᶠ) | O(B²) | Pairwise cosine similarity |
| Adaptive attention mechanism | O(BLNₕD²) | O(BLD²) | Multi-head attention |
| Energy-aware attention (dynamic) | O(BL⟨Nₕ⟩⟨D⟩²) | O(BL⟨D⟩²) | Reduced average complexity |
| Baseline GAN Architectures |  |  |  |
| Vanilla GAN | O(Bd) | O(B) | Generator + discriminator forward |
| WGAN-GP | O(Bd²) | O(Bd) | Gradient penalty computation |
| CTGAN | O(Bd + BK) | O(BK) | Mode-specific normalization |
| StyleGAN2-ADA | O(Bd² + BM²) | O(BM²) | Style modulation + mapping network |
| CycleGAN | O(2Bd + Bd) | O(Bd) | Dual generators + cycle consistency |
| Progressive GAN | O(Bd·P) | O(Bd) | P progressive stages |

**Table A7: Per-Component Training Overhead Breakdown**

| Component | Additional Time per Epoch | Percentage of Total | Overhead Factor | Justification |
| --- | --- | --- | --- | --- |
| Base GAN (generator + discriminator) | 3.28 min | 44.8% | 1.00× | Required baseline |
| Feature importance loss | +0.12 min | 1.6% | 1.04× | Single softmax computation |
| Distribution alignment loss | +0.08 min | 1.1% | 1.02× | Included in discriminator pass |
| Gradient regularization loss | +0.45 min | 6.1% | 1.14× | Second-order gradients |
| Clustering discriminator loss | +0.38 min | 5.2% | 1.12× | Pairwise distance O(B²) |
| Perturbation-aware loss (PGD) | +0.89 min | 12.2% | 1.27× | T=10 PGD iterations |
| Multi-scale preservation loss | +0.23 min | 3.1% | 1.07× | S=4 wavelet transforms |
| Diversity regularization loss | +0.31 min | 4.2% | 1.09× | Pairwise similarity O(B²) |
| Attention preservation loss | +0.18 min | 2.5% | 1.05× | Encoder + reconstruction |
| Standard attention mechanism | +1.84 min | 25.1% | 1.56× | Full 8-head attention |
| Adaptive attention (proposed) | +0.92 min | 12.6% | 1.28× | Dynamic head allocation |
| Energy monitoring overhead | +0.04 min | 0.5% | 1.01× | NVML queries |
| Total proposed overhead | +4.04 min | — | 2.23× | Sum of all components |
| Attention savings from adaptation | −0.92 min | −12.5% | 0.50× | 50% attention reduction |

**Table A8: Inference Latency Comparison (Milliseconds)**

| Method | Single Sample | Batch=32 | Batch=128 | Batch=512 | Throughput (samples/sec) |
| --- | --- | --- | --- | --- | --- |
| Vanilla GAN | 0.82 ± 0.05 | 2.14 ± 0.12 | 5.67 ± 0.31 | 18.92 ± 1.02 | 27,058 |
| WGAN-GP | 0.89 ± 0.06 | 2.31 ± 0.14 | 6.12 ± 0.35 | 20.45 ± 1.15 | 25,037 |
| CTGAN | 0.95 ± 0.07 | 2.48 ± 0.16 | 6.58 ± 0.38 | 21.98 ± 1.24 | 23,294 |
| StyleGAN2-ADA | 3.45 ± 0.21 | 8.92 ± 0.52 | 23.67 ± 1.35 | 79.12 ± 4.48 | 6,472 |
| CycleGAN | 1.78 ± 0.11 | 4.62 ± 0.28 | 12.25 ± 0.71 | 40.92 ± 2.32 | 12,514 |
| Progressive GAN | 2.34 ± 0.15 | 6.08 ± 0.37 | 16.12 ± 0.93 | 53.87 ± 3.05 | 9,505 |
| Proposed (full attention) | 2.12 ± 0.13 | 5.51 ± 0.33 | 14.62 ± 0.84 | 48.87 ± 2.77 | 10,476 |
| Proposed (adaptive attention) | 1.24 ± 0.08 | 3.22 ± 0.19 | 8.54 ± 0.49 | 28.54 ± 1.62 | 17,938 |
| Proposed (adaptive + INT8 benign) | 0.98 ± 0.06 | 2.55 ± 0.15 | 6.76 ± 0.39 | 22.59 ± 1.28 | 22,667 |

**Table A9: GPU Memory Consumption Comparison**

| Method | Training Memory (GB) | Inference Memory (GB) | Peak Memory (GB) | Memory Efficiency |
| --- | --- | --- | --- | --- |
| Vanilla GAN | 8.2 ± 0.3 | 2.1 ± 0.1 | 9.4 ± 0.4 | 1.00× (baseline) |
| WGAN-GP | 12.4 ± 0.5 | 2.3 ± 0.1 | 14.2 ± 0.6 | 0.66× |
| CTGAN | 6.8 ± 0.3 | 1.8 ± 0.1 | 7.8 ± 0.3 | 1.21× |
| StyleGAN2-ADA | 45.6 ± 1.8 | 8.9 ± 0.4 | 52.3 ± 2.1 | 0.18× |
| CycleGAN | 24.3 ± 1.0 | 5.2 ± 0.2 | 27.8 ± 1.1 | 0.34× |
| Progressive GAN | 34.2 ± 1.4 | 6.8 ± 0.3 | 39.2 ± 1.6 | 0.24× |
| Proposed (full attention) | 38.4 ± 1.5 | 7.2 ± 0.3 | 44.1 ± 1.8 | 0.21× |
| Proposed (adaptive attention) | 24.6 ± 1.0 | 4.8 ± 0.2 | 28.2 ± 1.1 | 0.33× |
| Proposed (memory optimized) | 18.9 ± 0.8 | 3.6 ± 0.2 | 21.7 ± 0.9 | 0.43× |

**Table A10: Computational Efficiency vs. Accuracy Trade-off Analysis**

| Method | Accuracy (%) | Training Time (h) | Inference (ms) | Acc/Train Time | Acc/Inference | Overall Efficiency Score |
| --- | --- | --- | --- | --- | --- | --- |
| Vanilla GAN | 84.23 | 7.8 | 0.82 | 10.80 | 102.72 | 0.67 |
| WGAN-GP | 91.67 | 11.3 | 0.89 | 8.11 | 103.00 | 0.72 |
| CTGAN | 87.23 | 6.6 | 0.95 | 13.22 | 91.82 | 0.68 |
| StyleGAN2-ADA | 93.45 | 48.1 | 3.45 | 1.94 | 27.09 | 0.45 |
| CycleGAN | 89.34 | 21.4 | 1.78 | 4.18 | 50.19 | 0.54 |
| Progressive GAN | 93.12 | 36.5 | 2.34 | 2.55 | 39.79 | 0.49 |
| Green-GAN-Security | 90.12 | 11.2 | 1.12 | 8.05 | 80.46 | 0.71 |
| Sustainable-IDS-GAN | 91.89 | 9.2 | 1.05 | 9.99 | 87.51 | 0.76 |
| Proposed (adaptive) | 98.73 | 10.9 | 1.24 | 9.06 | 79.62 | **0.89** |

**Table A11: Scalability Analysis Across Dataset Sizes**

| Dataset | Samples | Features | Proposed Training (h) | WGAN-GP Training (h) | Proposed Overhead | Scaling Factor |
| --- | --- | --- | --- | --- | --- | --- |
| NSL-KDD | 148,517 | 41 | 10.9 ± 0.6 | 11.3 ± 0.6 | 0.96× | 1.00× |
| UNSW-NB15 | 257,673 | 49 | 14.2 ± 0.7 | 15.8 ± 0.8 | 0.90× | 1.30× |
| CIC-IDS2017 | 2,830,743 | 78 | 48.6 ± 2.4 | 56.2 ± 2.8 | 0.86× | 4.46× |
| CIC-IDS2018 | 16,232,943 | 79 | 156.3 ± 7.8 | 189.4 ± 9.5 | 0.83× | 14.34× |
| Bot-IoT | 3,668,522 | 46 | 52.4 ± 2.6 | 58.7 ± 2.9 | 0.89× | 4.81× |
| CICDDOS2019 | 50,006,249 | 88 | 312.5 ± 15.6 | 398.2 ± 19.9 | 0.78× | 28.67× |
| CSE-CIC-IDS2018 | 6,226,100 | 80 | 78.9 ± 3.9 | 92.3 ± 4.6 | 0.85× | 7.24× |

**Table A12: Adaptive Attention Efficiency Analysis by Sample Category**

| Sample Category | Proportion | Attention Config | FLOPs (M) | Latency (ms) | Precision | Speedup vs Full |
| --- | --- | --- | --- | --- | --- | --- |
| High-threat (score > 0.7) | 8.2% | 8 heads, 512 dim | 134.2 | 1.89 | FP32 | 1.00× |
| Medium-threat (0.3-0.7) | 14.5% | 4 heads, 256 dim | 33.6 | 0.72 | FP16 | 2.63× |
| Low-threat (score ≤ 0.3) | 77.3% | 2 heads, 128 dim | 8.4 | 0.28 | INT8 | 6.75× |
| Weighted average | 100% | Dynamic | 24.8 | 0.47 | Mixed | **3.85×** |
| Full attention (baseline) | 100% | 8 heads, 512 dim | 134.2 | 1.89 | FP32 | 1.00× |

**Table A13: Overfitting Detection via Training-Validation Gap Analysis**

| Training Stage | Training Accuracy | Validation Accuracy | Gap | Overfitting Indicator |
| --- | --- | --- | --- | --- |
| Epoch 10 | 78.45 ± 1.23% | 77.89 ± 1.34% | 0.56% | No (gap < 2%) |
| Epoch 25 | 85.67 ± 0.98% | 84.92 ± 1.12% | 0.75% | No (gap < 2%) |
| Epoch 50 | 92.34 ± 0.67% | 91.45 ± 0.78% | 0.89% | No (gap < 2%) |
| Epoch 75 | 96.78 ± 0.52% | 95.67 ± 0.61% | 1.11% | No (gap < 2%) |
| Epoch 89 (convergence) | 98.89 ± 0.38% | 98.73 ± 0.41% | 0.16% | No (gap < 2%) |
| Epoch 100 | 99.12 ± 0.34% | 98.78 ± 0.39% | 0.34% | No (gap < 2%) |
| Epoch 125 | 99.34 ± 0.31% | 98.81 ± 0.38% | 0.53% | No (gap < 2%) |
| Epoch 150 | 99.45 ± 0.29% | 98.79 ± 0.37% | 0.66% | No (gap < 2%) |

**Table A14: Confusion Matrix for UNSW-NB15 Dataset**

| Actual \ Predicted | Normal | Generic | Exploits | Fuzzers | DoS | Recon | Analysis | Backdoor | Shellcode | Worms | Recall |
| --- | --- | --- | --- | --- | --- | --- | --- | --- | --- | --- | --- |
| Normal | 18,234 | 45 | 38 | 28 | 32 | 24 | 8 | 12 | 5 | 2 | 98.95% |
| Generic | 67 | 8,456 | 34 | 23 | 18 | 28 | 5 | 8 | 3 | 1 | 97.85% |
| Exploits | 45 | 28 | 6,789 | 34 | 23 | 19 | 12 | 7 | 4 | 2 | 97.51% |
| Fuzzers | 38 | 19 | 28 | 4,123 | 15 | 21 | 8 | 5 | 3 | 1 | 96.78% |
| DoS | 29 | 15 | 19 | 12 | 2,567 | 14 | 4 | 6 | 2 | 1 | 96.17% |
| Reconnaissance | 34 | 23 | 17 | 19 | 11 | 2,189 | 9 | 8 | 4 | 2 | 93.56% |
| Analysis | 12 | 8 | 14 | 9 | 5 | 11 | 378 | 4 | 2 | 1 | 85.14% |
| Backdoor | 18 | 11 | 9 | 7 | 8 | 9 | 3 | 289 | 3 | 2 | 80.50% |
| Shellcode | 8 | 4 | 6 | 4 | 3 | 5 | 2 | 2 | 187 | 1 | 84.23% |
| Worms | 5 | 2 | 3 | 2 | 2 | 3 | 1 | 1 | 1 | 28 | 58.33% |
| Overall Accuracy | — | — | — | — | — | — | — | — | — | — | **97.45%** |

**Table A15: Confusion Matrix for CIC-IDS2017 Dataset**

| Actual \ Predicted | Benign | DoS/DDoS | Brute Force | Web Attack | Botnet | Infiltration | Recall |
| --- | --- | --- | --- | --- | --- | --- | --- |
| Benign | 189,234 | 234 | 123 | 89 | 45 | 12 | 99.73% |
| DoS/DDoS | 312 | 45,678 | 67 | 34 | 23 | 8 | 98.96% |
| Brute Force | 89 | 45 | 2,345 | 28 | 12 | 5 | 92.91% |
| Web Attack | 67 | 28 | 34 | 1,567 | 18 | 7 | 91.03% |
| Botnet | 45 | 19 | 15 | 12 | 456 | 4 | 82.79% |
| Infiltration | 23 | 8 | 7 | 5 | 3 | 18 | 28.13% |
| Overall Accuracy | — | — | — | — | — | — | **98.12%** |

**Table A16: Confusion Matrix for CIC-IDS2018 Dataset**

| Actual \ Predicted | Benign | DoS | DDoS | Brute Force | Web Attack | Botnet | Infiltration | Recall |
| --- | --- | --- | --- | --- | --- | --- | --- | --- |
| Benign | 234,567 | 289 | 178 | 134 | 89 | 67 | 23 | 99.67% |
| DoS | 345 | 56,789 | 234 | 78 | 45 | 34 | 12 | 98.70% |
| DDoS | 267 | 312 | 78,456 | 89 | 56 | 45 | 18 | 99.01% |
| Brute Force | 123 | 67 | 78 | 3,456 | 45 | 28 | 8 | 90.77% |
| Web Attack | 89 | 45 | 56 | 34 | 2,123 | 23 | 12 | 89.13% |
| Botnet | 78 | 34 | 45 | 23 | 18 | 678 | 5 | 77.18% |
| Infiltration | 34 | 12 | 15 | 8 | 7 | 4 | 23 | 22.33% |
| Overall Accuracy | — | — | — | — | — | — | — | **97.89%** |

**Table A17: Minority Class Performance Summary Across All Datasets**

| Dataset | Minority Class | Training Samples | Test Samples | Precision | Recall | F1-Score | Augmentation Benefit |
| --- | --- | --- | --- | --- | --- | --- | --- |
| NSL-KDD | U2R | 52 | 52 | 67.16% | 86.54% | 75.63% | +27.92% vs baseline |
| NSL-KDD | R2L | 995 | 231 | 77.27% | 80.95% | 79.07% | +22.78% vs baseline |
| UNSW-NB15 | Worms | 174 | 48 | 56.00% | 58.33% | 57.14% | +31.45% vs baseline |
| UNSW-NB15 | Shellcode | 1,511 | 222 | 87.38% | 84.23% | 85.78% | +18.67% vs baseline |
| UNSW-NB15 | Backdoor | 2,329 | 359 | 82.34% | 80.50% | 81.41% | +15.23% vs baseline |
| CIC-IDS2017 | Infiltration | 36 | 64 | 32.73% | 28.13% | 30.25% | +19.56% vs baseline |
| CIC-IDS2017 | Heartbleed | 11 | 11 | 45.45% | 45.45% | 45.45% | +34.12% vs baseline |
| CIC-IDS2018 | Infiltration | 161 | 103 | 37.70% | 22.33% | 28.05% | +16.78% vs baseline |
| CIC-IDS2018 | Heartbleed | 15 | 15 | 53.33% | 53.33% | 53.33% | +38.67% vs baseline |
| Bot-IoT | Theft | 118 | 179 | 85.29% | 81.01% | 83.10% | +24.56% vs baseline |
| CSE-CIC-IDS2018 | Infiltration | 93 | 73 | 27.27% | 16.44% | 20.51% | +12.34% vs baseline |
| Average minority class | — | — | — | **56.48%** | **53.57%** | **53.61%** | **+23.83%** |
